# Transparent Gelation of Ionic Liquids Trapped in Silicone Microcup Structures under Scanning Electron Microscopy

**DOI:** 10.3390/gels9030179

**Published:** 2023-02-24

**Authors:** Kaede Iwasaki, Masayuki Okoshi

**Affiliations:** Department of Electrical and Electronic Engineering, National Defense Academy, 1-10-20 Hashirimizu, Yokosuka 239-8686, Japan

**Keywords:** gelation, ionic liquid, transparency, silicone rubber, microcup, scanning electron microscopy

## Abstract

It is expected that ionic liquids will be used in the future as electrolytes for electric double layer capacitors, but currently microencapsulation with a conductive or porous shell is required for their fabrication. Here, we succeeded in fabricating a transparently gelled ionic liquid trapped in hemispherical silicone microcup structures just by observing with a scanning electron microscope (SEM), which allows the microencapsulation process to be eliminated and electrical contacts to be formed directly. To see the gelation, small amounts of ionic liquid were exposed to the SEM electron beam on flat aluminum, silicon, silica glass, and silicone rubber. The ionic liquid gelled on all the plates, and a color change to brown was observed on all the plates except for silicone rubber. This change might be caused by reflected and/or secondary electrons from the plates producing isolated carbon. Silicone rubber could remove the isolated carbon due to the large amount of oxygen inside it. Fourier transform infrared spectroscopy revealed that the gelled ionic liquid included a large amount of the original ionic liquid. Moreover, the transparent, flat gelled ionic liquid could also be made into three-layer structures on silicone rubber. Consequently, the present transparent gelation is suitable for silicone rubber-based microdevices.

## 1. Introduction

In recent years, there has been a desire to develop energy storage devices for micropower supply to microdevices such as Internet of Things (IoT) devices [1]. Currently, energy storage devices are classified into heat storage and electric power storage, and the former has attracted attention as an approach in which phase change materials are microencapsulated with various shell materials [2,3]. For example, phase change materials as core materials include, but are not limited to, paraffin, n-octadecane, and n-hexadecane, and shell materials thereof include PMMA, SiO_2_, Poly(St-MMA), Fe_3_O_4_-polyurea, Fe_3_O_4_-PMMA, and cellulose acetate [4,5,6,7,8,9]. On the other hand, the main approach to electric power storage is the formation of chemical batteries and electric double layer capacitors, but there are still few studies on microencapsulation. Recently, however, some research on microencapsulation of ionic liquids as electrolytes with a conductive or porous shell material has been reported [10,11,12], and the possibility of the formation of electric power storage devices in micron size has been mentioned. In this case, the shell for microencapsulation is required to be a conductive or porous material to make electrical contact with the outside. Therefore, if an ionic liquid can be trapped in a certain microcup and area-selectively gelled, it will be possible to form a microelectric double layer capacitor that can make direct electrical contacts with the ionic liquid.

In this paper, we drop a small amount of ionic liquid into hemispherical silicone microcup structures fabricated by 193 nm ArF excimer laser-induced photodissociation of Si-O-Si bonds of silicone rubber [13]. When the ArF excimer laser irradiates the silicone rubber surface, the main chain of Si-O-Si bonds of silicone rubber can be photodissociated into the lower molecules, resulting in microswelling of the laser-irradiated area. Periodically, we use silica glass microspheres of 2.5 μm diameter, which cover the entire surface of silicone rubber during laser irradiation. Each swelled silicone rubber piece underneath the microspheres shows a truncated cone shape in micron size. A little later, lower molecular weight silicone pieces, which are generated by photodissociation, might be ejected from the microswelled silicone rubber during laser irradiation along the curvature of each microsphere. After removing the microspheres, hemispherical silicone microcups on the microswelling structures of silicone rubber can be obtained. Then, the trapped ionic liquid is found to be gelled just by observing with a scanning electron microscope (SEM), which allows the microencapsulation process to be eliminated and electrical contacts to be formed directly. There are previous reports on the phenomenon of ionic liquids being solidified by exposure to electron beams [14,15,16], but the original points of this paper can still be asserted as follows: (1) gelation of ionic liquid trapped in hemispherical silicone microcup structures just by observing with SEM; (2) transparent gelation only on silicone rubber compared with other conductive, semiconducting, and insulating plates; (3) maintaining most of the chemical bonding state after gelation due to a high content of the original ionic liquid; (4) a flat multilayer structure of transparently gelled ionic liquid on periodic microswelling structures of silicone rubber.

Moreover, since the surface of the sample, in which the ionic liquid is trapped in a part of the hemispherical silicone microcup structures, exhibits a superhydrophobic property [13], an air gap layer is formed on the surface of the silicone microcup structures to protect the structure even if it is immersed in water [17,18,19]. Therefore, as for future prospects, it is considered possible to realize a microenergy storage device that can supply minute power to IoT devices, which can be used underwater [20].

## 2. Experimental Method

**Figure 1:**
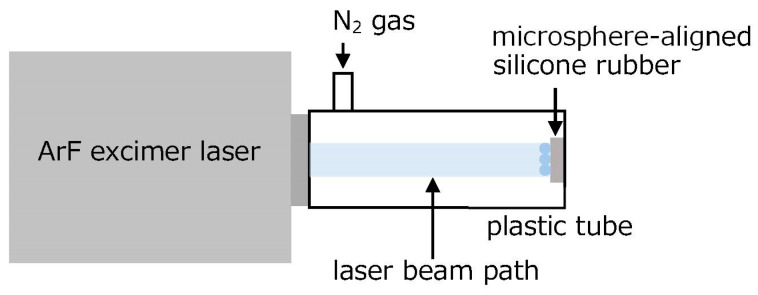
Schematic drawing of the experimental setup for fabricating the silicone microcup structures.

Figure 1 shows a schematic drawing of the experimental setup for fabricating the silicone microcup structures [13]. Silica glass microspheres of 2.5 µm diameter (Nippon Shokubai KE-P250) were dispersed in ethanol, and the dispersed solution was dripped onto the surface of a 2-mm-thick silicone rubber. Thus, a single layer of silica glass microspheres was formed on the silicone rubber after air drying, in addition to removal of excess microspheres by wiping. The sample was placed approximately 80 mm away from the outlet of an ArF excimer laser (COMPexPro110, Coherent, PA, USA), as shown in Figure 1. The laser beam path was filled with N_2_ gas at a flow rate of 5 L/min to avoid strong optical absorption of oxygen molecules in the air. The 80 mm setting was experimentally convenient and showed that N_2_ gas flow is necessary if the laser beam path is long. The ArF excimer laser irradiated the sample surface without a lens. The irradiation area was typically 10 × 10 mm^2^. A single pulse fluence of the ArF excimer laser was approximately 30 mJ/cm^2^.

The pulse repetition rate and pulse number were 1 Hz and 1800, respectively. All the laser irradiations were carried out at room temperature. After the laser irradiation, silica glass microspheres were removed by a 1 wt% HF chemical etching for 30 s.

An ionic liquid of 1-butyl-3-methylimidazolium bis(trifluoromethanesulfonyl)imide (Kanto chemical), which shows an extremely low vapor pressure, was used. We chose this as it is a typical ionic liquid that is transparent and easy to handle. Then, the ionic liquid was dropped onto the fabricated silicone microcup structures. After that, the droplets of ionic liquid were successfully trapped in the silicone microcup structures and were exposed to the electron beam emitted for SEM (Pro, Phenomworld, MA, USA) observation. To see the gelation of ionic liquid, we observed the fluidity and color change of ionic liquid dropped onto flat aluminum (Al), silicon (Si), slide glass, and silicone rubber plates after the exposure to the electron beam. To inspect the chemical bonding state of the gelled ionic liquid, as a substrate, we fabricated periodic microswelling structures of silicone rubber to obtain a gelled ionic liquid that is flat and transparent on at least one side. Specifically, during the formation of the silicone microcup structures, the silicone microcups were removed at the same time as the silica glass microspheres were removed by increasing the etching time of 1 wt% HF aqueous solution from 30 to 90 s. As a result, the periodic microswelling structures of silicone rubber formed under the silicone microcups remained. Then, the chemical bonding state of the gelled ionic liquid was analyzed by Fourier transform infrared spectroscopy (FT-IR, FT/IR-610, Jasco, Tokyo, Japan) using the attenuated total reflection (ATR) method. Moreover, on the periodic microswelling structures of silicone rubber, a flat multilayer structure of transparently gelled ionic liquid was fabricated for further applications.

## 3. Results and Discussion

**Figure 2:**
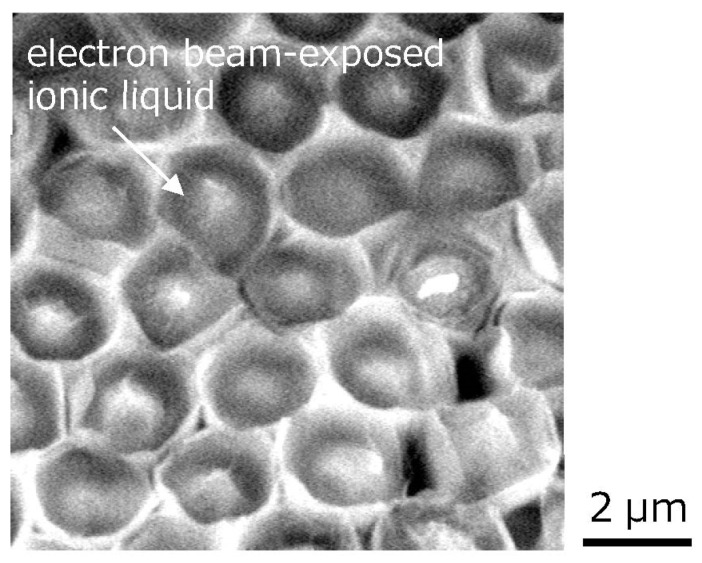
SEM image of the electron beam-exposed ionic liquids trapped in the hemispherical silicone microcup structures.

Figure 2 shows the SEM image of the ionic liquids trapped in the silicone microcup structures fabricated by the ArF excimer laser. A hemispherical silicone microcup with a diameter of approximately 2.5 μm corresponding to the diameter of the silica glass microsphere was fabricated on each silicone microswelling structure, with a height of approximately 1 μm [13]. The trapped ionic liquid appeared to lose its fluidity after the long SEM observation for 15 to 30 min. During that time, the ionic liquids slightly contracted in volume.

**Figure 3:**
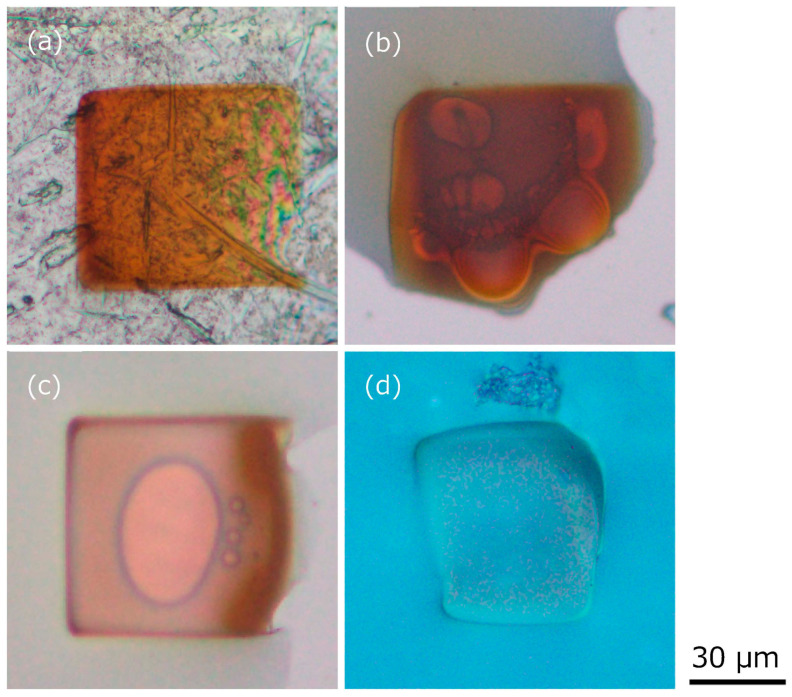
Small amounts of the ionic liquid dropped onto various flat plates of (**a**) Al, (**b**) Si, (**c**) silica glass, and (**d**) silicone rubber after exposure to electron beam through SEM observation.

To visually observe the fluidity and color change of the ionic liquid exposed to the electron beam emitted for SEM observation, the ionic liquid was dropped onto four types of flat plates and exposed to the electron beam through SEM observation, as shown in Figure 3. The electron beam exposure conditions were 15 kV and 30 min. The thickness of the dropped ionic liquid was roughly several microns. In the case of Al as a conductor (which is the stage for SEM observation and is grounded), Si as a semiconductor, a slide glass as an insulator, and silicone rubber (these are placed on the Al stage via conductive tape), the ionic liquid lost its fluidity and appeared gel-like rather than completely solid. Also, a significant color change of the ionic liquid to brown was observed on Al, Si, and silica glass. On the other hand, no significant color change of the ionic liquid was observed on silicone rubber. In all cases, it was also found that bubbles were generated at the interface between the plates and the ionic liquid immediately after exposure to the electron beam. Thus, loss of fluidity and color change are not considered to be strongly related. The cause of the color change will be discussed later. With our benchtop SEM equipment, we were unable to determine the current. Also, the voltage varied between 5, 10, and 15 kV. In both cases, the ionic liquid lost its fluidity after electron beam exposure. Experiments below 5 kV were not performed, and the minimum voltage for gelation was not determined. Therefore, for the ionic liquid on the silicone rubber, which did not change color, a polymerization reaction of carbon chains of cation also might have occurred due to the exposure to the electron beam [16] and gelation with the generation of gas. It is considered relatively useful in terms of application for the ionic liquid to lose fluidity in a transparent state and enter a solidified state such as gelation.

**Figure 4:**
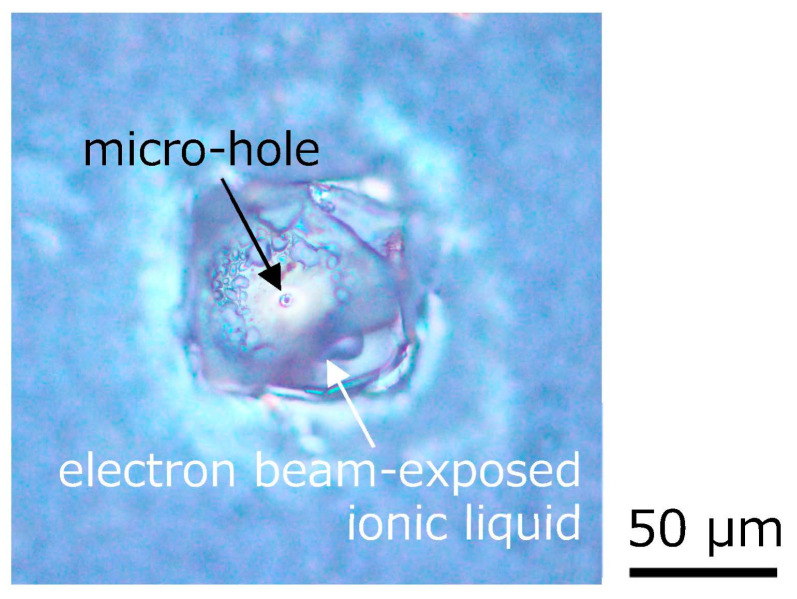
A microhole formed on the electron beam-exposed ionic liquid on silicone rubber by a 532 nm CW laser.

Figure 4 shows the optical microscopic photograph of the microhole formed on the electron beam-exposed ionic liquid on silicone rubber by a continuous wave (CW) laser with a wavelength of 532 nm, which was focused by a microscope objective lens and irradiated for approximately 1 min. A clear microhole was formed in the laser-irradiated area. Also, no remarkable outflow of the ionic liquid from the vicinity of the microhole was observed. In addition, the microhole did not disappear over time. The many fine bubbles around the formed microhole are caused by gas generated at the interface between the ionic liquid and silicone rubber by electron beam exposure. Therefore, it is considered that the inside of the electron beam-exposed ionic liquid was also partially polymerized. Since the microhole is formed by the thermal effect of the laser, however, it is thought that the microhole is thermally modified, and as a result, the ionic liquid inside may not leak.

**Figure 5:**
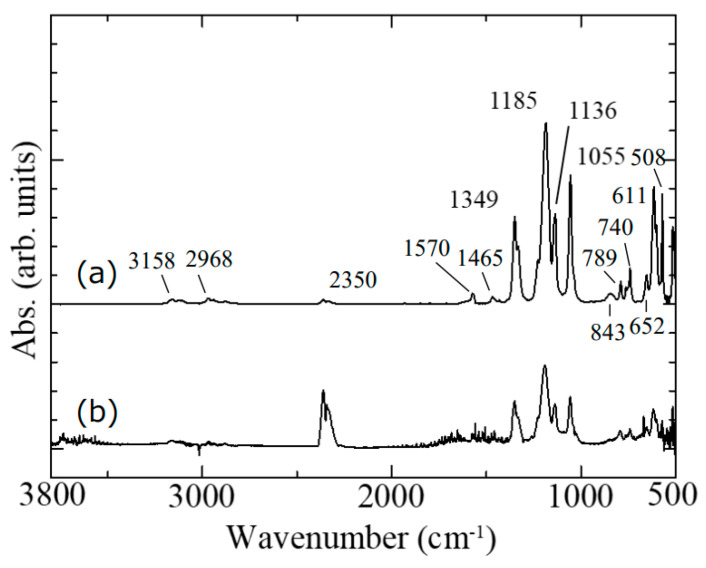
ATR-FT-IR spectra of (**a**) original ionic liquid and (**b**) electron beam-exposed ionic liquid.

**Figure 6:**
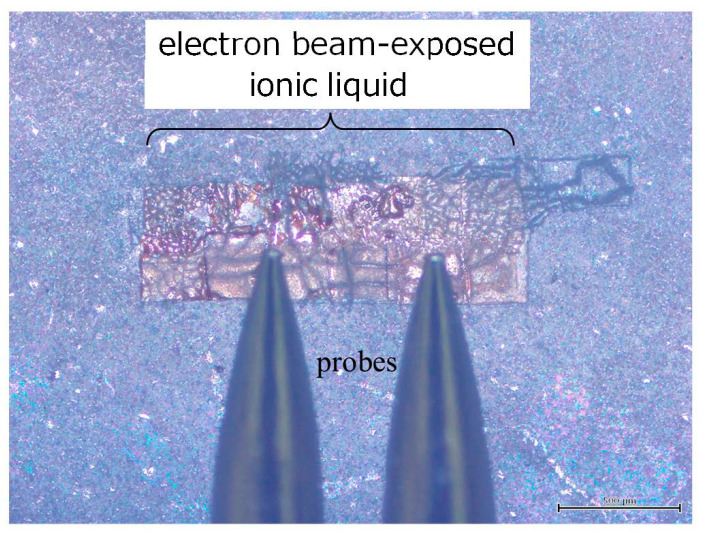
Optical microscopic photograph of the electron beam-exposed ionic liquid with a relatively wide area of 500 × 1500 μm^2^.

The chemical bonding state of the electron beam-exposed ionic liquid was measured by ATR-FT-IR, as shown in Figure 5. The original ionic liquid was also measured for comparison. In the ATR method, a Ge prism was used and the spectrum of one reflection was measured at an incident angle of 45 degrees. The ionic liquid and the prism were brought into direct contact. For the measurement, we considered forming an electron beam-exposed ionic liquid with a flat surface and a wide area. If the substrate is flat, the electron beam-exposed ionic liquid will not be flat due to the surface tension of the ionic liquid. Thus, we used silicone rubber with periodic microswelling structures as the substrate [17]. Specifically, during the formation of the silicone microcup structures, the silicone microcups were removed at the same time as the silica glass microspheres were removed by increasing the etching time of 1 wt% HF aqueous solution from 30 to 90 s. As a result, the periodic microswelling structures of silicone rubber formed under the silicone microcups remained. On the other hand, in order to obtain a sample with a large area, the electron beam-exposed area of 60 × 60 μm^2^ was scanned to obtain a sample with a relatively wide area of 300 × 300 μm^2^ in total. The thickness of the electron beam-exposed area was roughly 1 μm. As a practical example of large-area enlargement, a 250 × 250 μm^2^ electron beam-exposed area was scanned 12 times to obtain a relatively large area sample of 500 × 1500 μm^2^ in total, as shown in Figure 6. A pair of probes was able to contact the electron beam-exposed ionic liquid directly, indicating the possibility of investigating the electrical properties of the sample as well in the future. Compared with the case of the original ionic liquid (Figure 5a), the overall spectral intensity decreased, but no change was observed in the absorption spectrum even after careful washing of the sample (Figure 5b). In this case, the peak at 2350 cm^−1^ is due to atmospheric CO_2_ in the path of the IR light. The ATR prism made almost perfect contact with the original ionic liquid. On the other hand, the electron beam-exposed ionic liquid did not make complete contact with the prism due to its surface roughness, and it is considered that the IR absorption by CO_2_ in the air was enhanced due to the micron-sized air gaps. Thus, the ATR-FT-IR spectra revealed that the electron beam-exposed ionic liquid remained almost unchanged compared with the original structure. This means that a large amount of the original ionic liquid is included in the electron beam-exposed ionic liquid. In addition, the difference between before and after electron beam exposure was also examined by microscopic Raman spectroscopy (NRS-5100, Jasco, Tokyo, Japan). Although the Raman spectrum of the original ionic liquid could be measured [20], in the case of the electron beam-exposed ionic liquid, it was necessary to reduce the output power of the 532 nm CW laser as much as possible, but even then, microholes were formed and fluorescence was also generated, and as a result the spectrum could not be measured.

**Figure 7:**
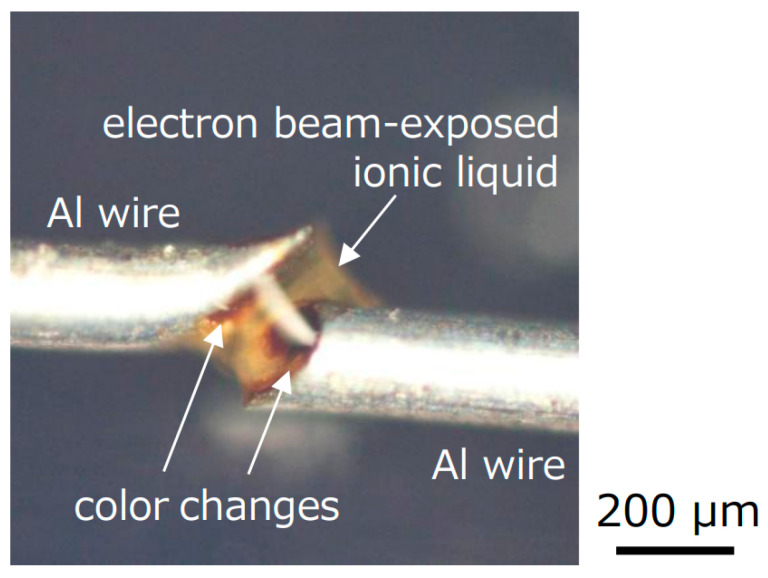
Photograph of the ionic liquid in the gap between Al wires after electron beam exposure.

To investigate the cause of the color change of the ionic liquid after electron beam exposure, an ionic liquid was dropped in the gap between Al wires of 0.2 mm diameter, and an electron beam was irradiated, as shown in Figure 7. The two Al wires were suspended. During the electron beam exposure, a color change to brown started to occur from the ends of both Al wires. On the other hand, no significant color change was observed in the center of the ionic liquid between Al wires, even when the gelation occurred. Thus, it is considered that the color change starts to occur from the surface of plates in Figure 3 as well, which might be caused by reflected and/or secondary electrons from the plates. Considering the disturbing fluorescence generation during Raman spectroscopy that we mentioned above, carbon chains of cation in the ionic liquid might be partly decomposed leading to the production of isolated carbon, resulting in the color change to brown. In fact, when a silicone rubber plate was used, the color of the ionic liquid almost did not change. Since silicone rubber includes a large amount of oxygen molecules, oxygen molecules and/or atoms might be ejected from the silicone rubber during electron beam exposure to remove the isolated carbon, resulting in the successful transparent gelation of ionic liquid only on the silicone rubber.

**Figure 8:**
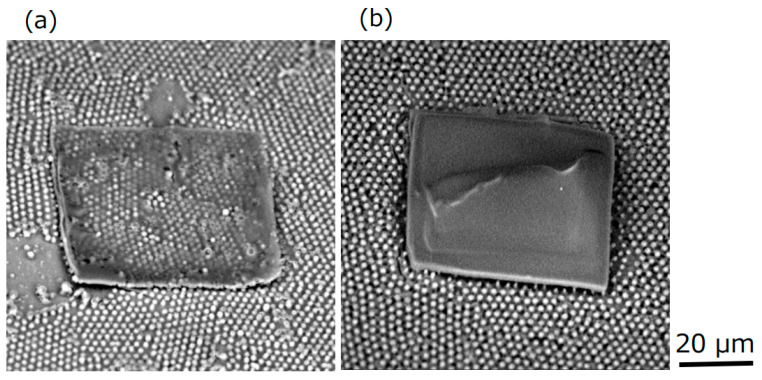
SEM images of the transparent, flat gelled ionic liquid on the periodic microswelling structures of silicone rubber: (**a**) one layer and (**b**) three layers.

Based on the present results, it was possible to form an ionic liquid that gelled transparently and evenly on silicone rubber with periodic microswelling structures. If this gelled ionic liquid with a film thickness of approximately 1 μm can be layered, the electrolyte in the region where the microcup exists on the same silicone rubber will be gelled in a hemispherical shape in a minute region, and the transparent, flat gelled ionic liquid will be formed at the same time with a desired film thickness in the region where the microcup is removed. Then, the possibility of new applications of ionic liquids only on silicone rubber is expected. Thus, the ionic liquid was dropped onto the silicone rubber on which the periodic microswelling structures had formed, and the flattened ionic liquid was exposed to the electron beam. After that, the dropping of the ionic liquid and electron beam exposure were repeated, and finally the unexposed portion was removed with water. Figure 8 shows the SEM images for one layer and for three layers. It was found that it is possible to form layered structures. As for future prospects, by incorporating an electroluminescent material into a transparent, flat gelled ionic liquid, as the gelled ionic liquid is expected to have an ionic conduction, it will be possible to fabricate a flat microlight source on silicone rubber with micropower storage functionality, and to develop new microdevices.

## 4. Conclusions

A transparently gelled ionic liquid trapped in hemispherical silicone microcup structures was successfully fabricated just by observing with SEM. To see the gelation, small amounts of ionic liquid were dropped onto flat Al, Si, silica glass, and silicone rubber plates, and the electron beam for SEM was irradiated. The ionic liquids gelled on all the plates, and a color change to brown was observed on all the plates except for the silicone rubber. The color change should be separate from the gelation; it might be caused by reflected and/or secondary electrons from the plates producing isolated carbon in the gelled ionic liquid. Since the silicone rubber includes a large amount of oxygen molecules, the isolated carbon could be almost completely removed by oxygen molecules and/or atoms ejected from the silicone rubber during the electron beam exposure. The ATR-FT-IR spectra reveal that the gelled ionic liquid includes a large amount of the original ionic liquid. Moreover, the ionic liquid could be thinly flattened on periodic microswelling structures of silicone rubber and could gel. Thus, the transparent, flat gelled ionic liquid could also be made into three-layer structures. Therefore, the gelation of ionic liquid by observing with SEM is suitable for silicone rubber-based microdevices. For microelectric power storage applications, the present results allow the microencapsulation process to be eliminated and electrical contacts to be formed directly for the formation of microelectric double layer capacitors. In addition, by forming a gelled ionic liquid trapped in silicone microcup structures and a transparent, flat gelled ionic liquid on the same silicone rubber, it is possible to fabricate a new microdevice that simultaneously has a planar microlight source and microelectric power storage.

## Data Availability

The data that support the findings of this study are available from the corresponding author, M.O., upon reasonable request.
